# Chronobiological Patterns of Aneurysmal Subarachnoid Hemorrhage in Central China

**DOI:** 10.5334/gh.1117

**Published:** 2022-04-28

**Authors:** Yuehui Wu, Nan Tang, Liangtao Xia, Tianyu Liu, Hao Yu, Xiaobing Jiang, Xinyu Yu

**Affiliations:** 1 Department of Neurosurgery, Union Hospital, Tongji Medical College, Huazhong University of Science and Technology, Wuhan 430022, CN; 2 Department of Neurosurgery, The Affiliated Hospital of Guizhou Medical University, Guizhou, Guiyang, P. R. China, CN; 3 Division of Cardiothoracic and Vascular Surgery, Tongji Hospital, Tongji Medical College, Huazhong University of Science and Technology, Wuhan 430030, CN

**Keywords:** Aneurysmal subarachnoid hemorrhage, time series, chronobiological pattern, incidence, rhythmicity

## Abstract

**Background:**

Aneurysmal subarachnoid hemorrhage (aSAH) is an acute and sometimes fatal cerebrovascular disease. The chronobiological patterns of aSAH are still unclear worldwide. This 15-year time-series study aims to clarify the chronobiological patterns including seasonal, monthly, weekly, and circadian distributions of aSAH.

**Methods:**

We retrospectively analyzed the medical records of aSAH patients in central China. To investigate seasonal and weekly distributions, we used the χ2 goodness-of-fit test to analyze the uniformity of the onset time. To explore monthly and circadian distributions, we established Fourier models to show the rhythmicity in chronobiological patterns. Subgroup analyses were conducted to assess the impact of age, gender, hypertension statuses, and aneurysmal characteristics (number, size, and location) on the chronobiological patterns of aSAH.

**Results:**

A total of 1469 patients with aSAH were recruited in the study. The seasonal and monthly distribution exhibited significantly higher incidence in winter and January/December and lower incidence in summer and July. The weekly distribution of aSAH onset showed no significant uneven variation. The circadian distribution of aSAH exhibited a significant pattern (p = 0.0145), with a morning peak around 8:00, and a late afternoon peak at 16:00–20.00. The circadian rhythmicity varied in subgroups of different ages, genders, and aneurysmal locations.

**Conclusion:**

The occurrence of aSAH exhibits significant circannual and circadian patterns among the Chinese population. Patients with aSAH of different ages, genders, and aneurysmal locations would present different chronobiological patterns.

## 1. Introduction

Aneurysmal subarachnoid hemorrhage (aSAH) is an acute and sometimes fatal cerebrovascular event, and the aggregate worldwide incidence is around 10.5 cases per 100,000 person-years [1]. The average case fatality rate for aSAH is 50% and approximately 30% of survivors need lifelong care [1]. Although the incidence of aSAH is much lower than that of ischaemic stroke and intracerebral hemorrhage, the annual number of deaths from aSAH is similar to the other two types of stroke [2]. Previous studies suggested that the onset of aSAH occurred heterogeneously at different periods and occurred more frequently in winter and early morning [3,4]. Determining the specific periods with a high incidence of aSAH could provide clues for identifying triggering factors, and formulating new preventive strategies for this devastating disease.

However, the chronobiological patterns including seasonal, monthly, weekly, and circadian distributions of aSAH remain nebulous. Previous studies of different races yielded inconsistent results on the diurnal variation of aSAH [3,5,6]. Most of them were conducted in Western countries, and they lacked subgroup analyses of influencing factors such as age, gender, and hypertension [5,6,7,8,9,10,11]. The incidence of aSAH onset could be very different among people in distinct cultures for differential lifestyle habits. Thus, we performed this 15-year time-series study in central China to clarify the chronobiological patterns of aSAH and to expound on the temporal trends of aSAH onset.

## 2. Methods

### 2.1 Study design

The purpose of this study was to determine whether there is a particular seasonal, monthly, weekly, and circadian variation of the occurrence of aSAH. We divided patients into different subgroups according to 4 seasonal periods, 12 1-month periods, and 7 1-day-of-the-week periods according to their onset date. The climate condition in central China divided four seasons into March to May as Spring, June to August as Summer, September to November as Autumn and December to February as Winter. If the medical records of patients reported the exact time point of aSAH onset in a day, they were included in the analysis of the circadian rhythm. The onset time in one day was categorized into 24 1-hour increments (eg, onset time between 12:00 to 12:59 were recorded as the 12:00 group). Subgroup analyses were performed to further investigate whether differences in sex, age, size of the ruptured aneurysm (≥7 mm or <7 mm), location of the ruptured aneurysm, number of aneurysms (multiple or single), and comorbidity would affect the chronobiological patterns of aSAH. We reported this study following the Strengthening the Reporting of Observational Studies in Epidemiology checklist.

### 2.2 Patient inclusion and data extraction

We screened all the electronic medical records in Wuhan Union hospital from 2005 to 2020, and we recruited confirmed cases of aSAH. Wuhan Union hospital is a major comprehensive hospital in central China that mainly treats patients from Hubei Province. We primarily used the International Classification of Diseases-10 codes (I60.901) to identify patients diagnosed with subarachnoid hemorrhage from 1 January 2005 to 1 August 2020. All patients diagnosed with SAH were enrolled and their imaging information (ie, CT, CT angiography, digital subtraction angiography, and magnetic resonance angiography) and surgical records were checked for further confirmation. Patients with saccular intracranial aneurysms (IA) could be identified by imaging information or surgical records were included for subsequent analyses. If the SAH of patients was caused by intracerebral hemorrhage, pseudoaneurysm, moyamoya disease, arteriovenous malformations, trauma, and neoplasms, they were excluded in this study. We also excluded patients with recurrent aSAH. We separately extracted the following information of included patients: age, gender, comorbidities, number of aneurysms, size of the ruptured aneurysm (the largest diameter of IAs), and location of the ruptured aneurysm. The locations of ruptured IAs were categorized into three subsets for subsequent analyses [12,13]: a) IA in anterior circulation arteries after bifurcation of the internal carotid artery (Ant-IA group); b) IA in internal carotid artery and branches except for Ant-IA (ICA-IA group); c) IA in posterior circulation artery (Post-IA group). The exact onset time of aSAH was inferred from the abrupt occurrence of typical symptoms (severe headache, vomit, and loss of consciousness) based on electronic medical records.

### 2.3 Statistical analysis

Continuous variables are presented as mean±SD or IQR, whereas categorical variables are described by frequency or percentage. The seasonal and weekly distribution of aSAH onset was tested for uniformity by the χ^2^ goodness-of-fit test. A χ^2^ value large enough to reject the hypothesis implied nonuniformity. To complete the test, we set values following uniform distribution as the expected values. To investigate the monthly trends and rhythmicity, we used a one harmonic Fourier model of 12 months to fit our data. For the circadian variation, the analysis of rhythmicity was performed by applying a four-harmonics Fourier model (periods of 24, 12, 8, and 6 hours) according to the previous research [14]. The goodness of fit of the Fourier model was represented by the calculated R square. We compared the nonlinear Fourier model fitting with straight-line model fitting and the F statistic and p-value was reported to represent the statistical significance of the fitted result, with a P-value less than 0.05 considered significant. We used GraphPad Prism V.8.3.0. to complete all the statistical analyses.

## 3. Results

### 3.1 Basic characteristics of the study population

**Figure 1:**
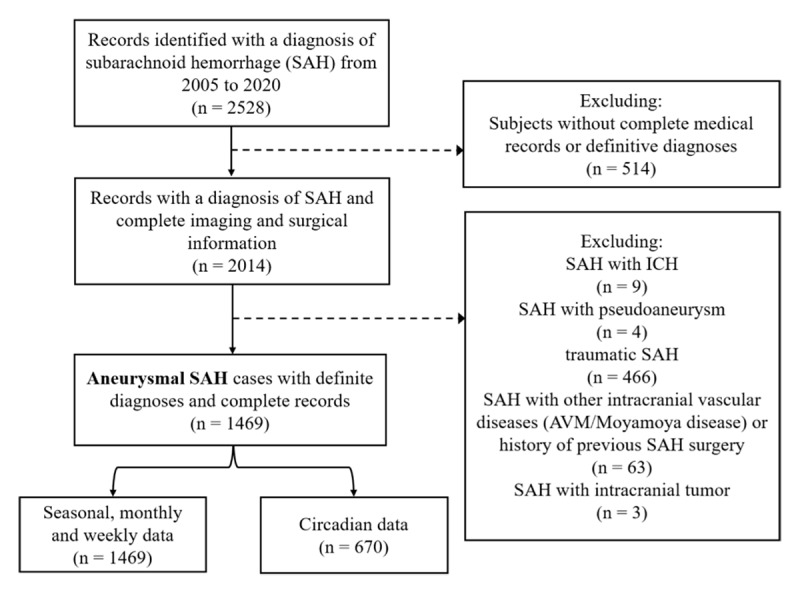
Flowchart of the study population selection. ICH, intracerebral hemorrhage; AVM, arteriovenous malformations.

A total of 1469 patients with a diagnosis of aSAH were recruited (***Figure 1***), including 545 (37.1%) males and 924 (62.9%) females. The mean age was 54.7±10.0 years, and 727 (49.5%) patients were older than 55 years old. A prior history of hypertension was present in nearly half of patients (40.8%), whereas diabetes mellitus occurred in a minority (3.6%) (Table S1). The detailed characteristics of intracranial aneurysms are shown in Table S2. Among the study population, 670 patients had an exact onset time of aSAH, and we included them in the circadian analysis (see Table S3 and S4).

### 3.2 Seasonal and monthly variation of aSAH onset

**Figure 2:**
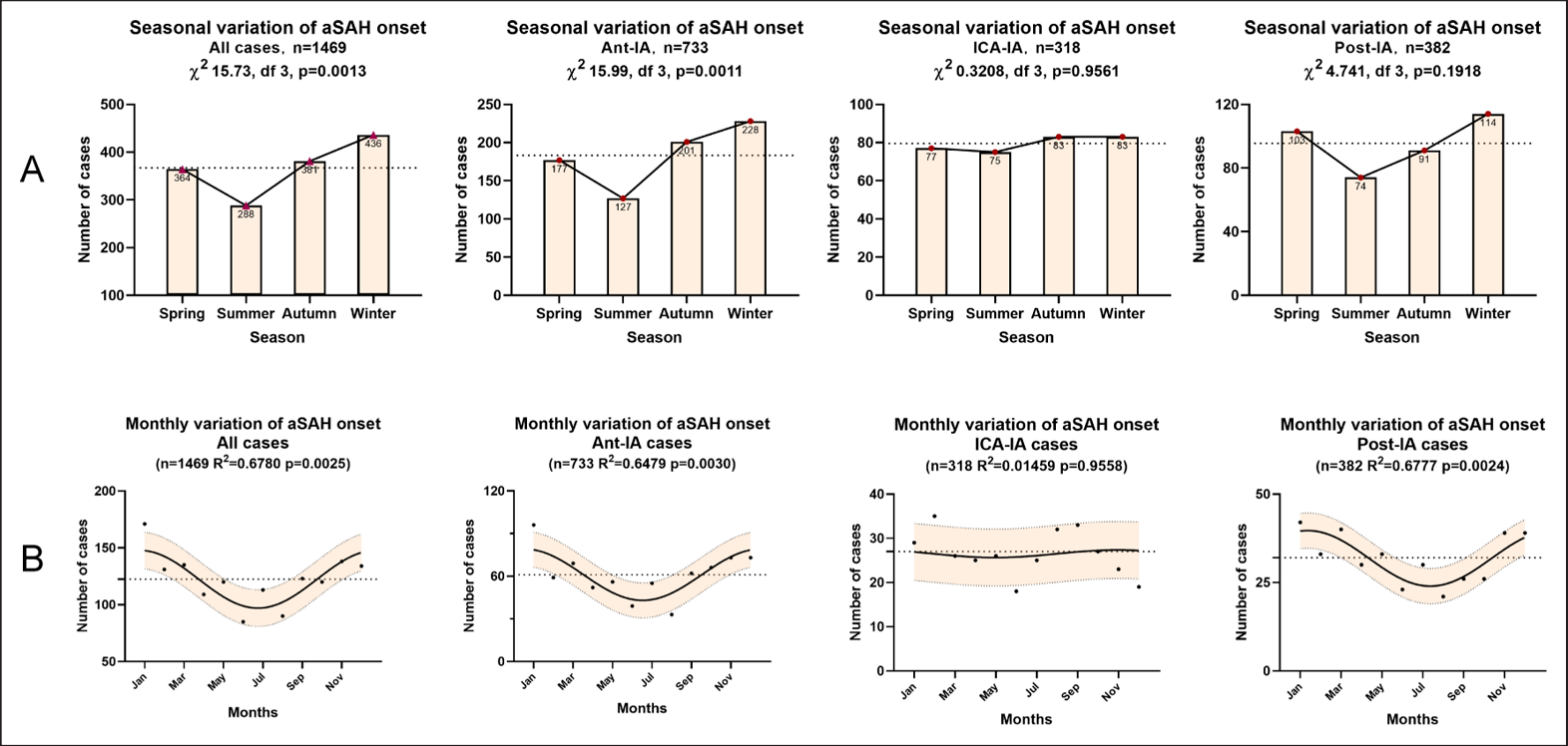
Circannual analyses of aSAH onset. A) Seasonal distributions of aSAH onset, tested by the χ2 goodness-of-fit test. B) Monthly variations of aSAH onset, fitted by the one harmonic Fourier model with a period of 12 months. The solid line is the calculated best-fitting curve. The filled area represents 95% CI. The dotted line represents the midline estimated statistic of rhythm. aSAH, aneurysmal subarachnoid hemorrhage; Ant-IA, aneurysm in anterior circulation arteries after bifurcation of the internal carotid artery; ICA-IA, aneurysm in internal carotid artery and branches except for Ant-IA; Post-IA, aneurysm in posterior circulation artery.

A significant temporal pattern was observed in the seasonal distribution of aSAH onset (χ^2^ 15.73, df, 3, p = 0.0013) (***Figure 2A***). Among the study population, most cases occurred in winter (436 cases, 29.68%) and the fewest cases occurred in summer (288 cases, 19.61%). This pattern also existed in the Ant-IA group (p = 0.0011), with 228 cases (31.11%) occurred in winter and 127 cases (17.33%) occurred in summer. For the Post-IA group, the peak frequency also existed in winter (114 cases, 29.84%) and the lowest frequency existed in summer (74 cases, 19.37%), but there is no statistically significant difference (p = 0.1918). For the ICA-IA group (91.8% cases had aneurysm located in the internal carotid), the frequencies in each season were similar, with no significant variation (p = 0.9561). Other subgroups also showed higher incidence in winter and lower incidence in summer (Figure S1).

On the other hand, we used a non-linear Fourier model to analyze the monthly variation of aSAH onset. A significant temporal pattern was identified by the model (p = 0.0025), with a peak around January/December and a nadir around July (***Figure 2B***). We observed this pattern in all subgroups (Figure S2) except for the ICA-IA group (p = 0.9558) (***Figure 2B***).

### 3.3 Weekly distribution of aSAH onset

**Figure 3:**
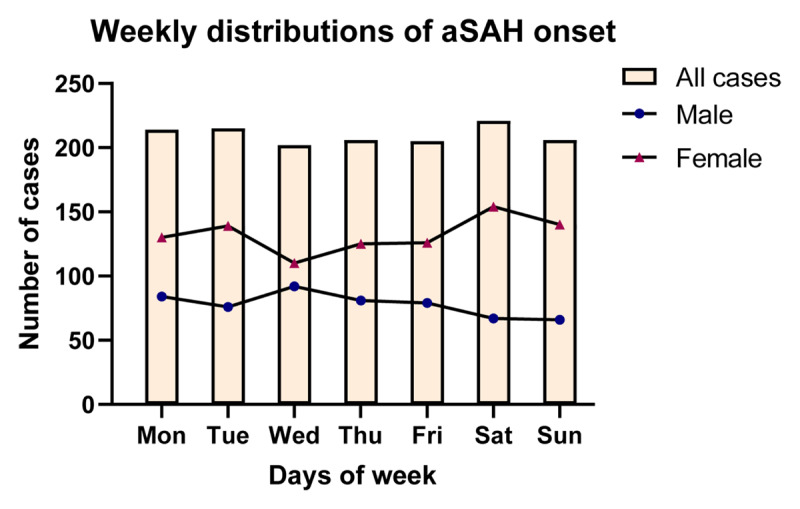
Weekly distributions of aSAH onset of all cases, male cases, and female cases. aSAH, aneurysmal subarachnoid hemorrhage.

There was no significant uneven variation in the weekly distribution of aSAH onset of total population (χ^2^ 0.6695, df 6, p = 0.9951), male patients (χ^2^ 3.298, df 6, p = 0.7707) and female patients (χ^2^ 4.454, df 6, p = 0.6155) (***Figure 3***). Analyses in other subsets also showed no uneven variation (Table S5).

### 3.4 Circadian variation of aSAH onset

**Figure 4:**
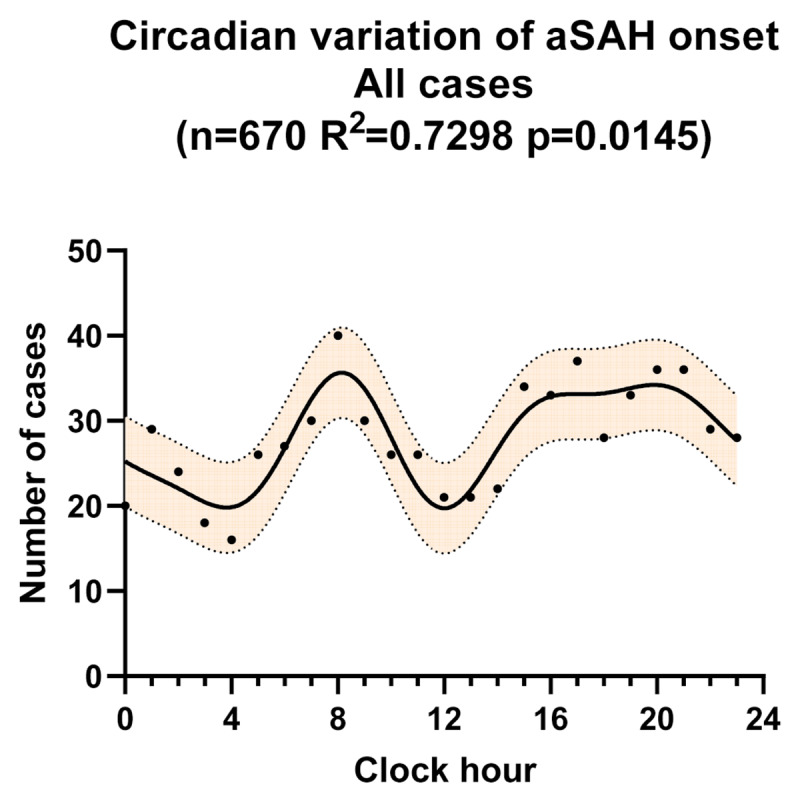
Circadian variation of aSAH onset for all cases, fitted by a four-harmonic Fourier model with periods of 24, 12, 8, and 6 hours. The solid line is the calculated best-fitting curve. The filled area represents 95% CI. aSAH, aneurysmal subarachnoid hemorrhage.

Non-linear Fourier rhythm analysis identified a significant circadian pattern in the occurrence of aSAH in the overall population (p = 0.0145), with two peaks and two troughs (***Figure 4***). A narrow peak was observed in the morning around 8:00 and a wider peak was observed in the late afternoon in 16:00–20:00. One nocturnal trough appeared in 3:00–4:00 and the other trough appeared at midday in 12:00–13:00.

**Figure 5:**
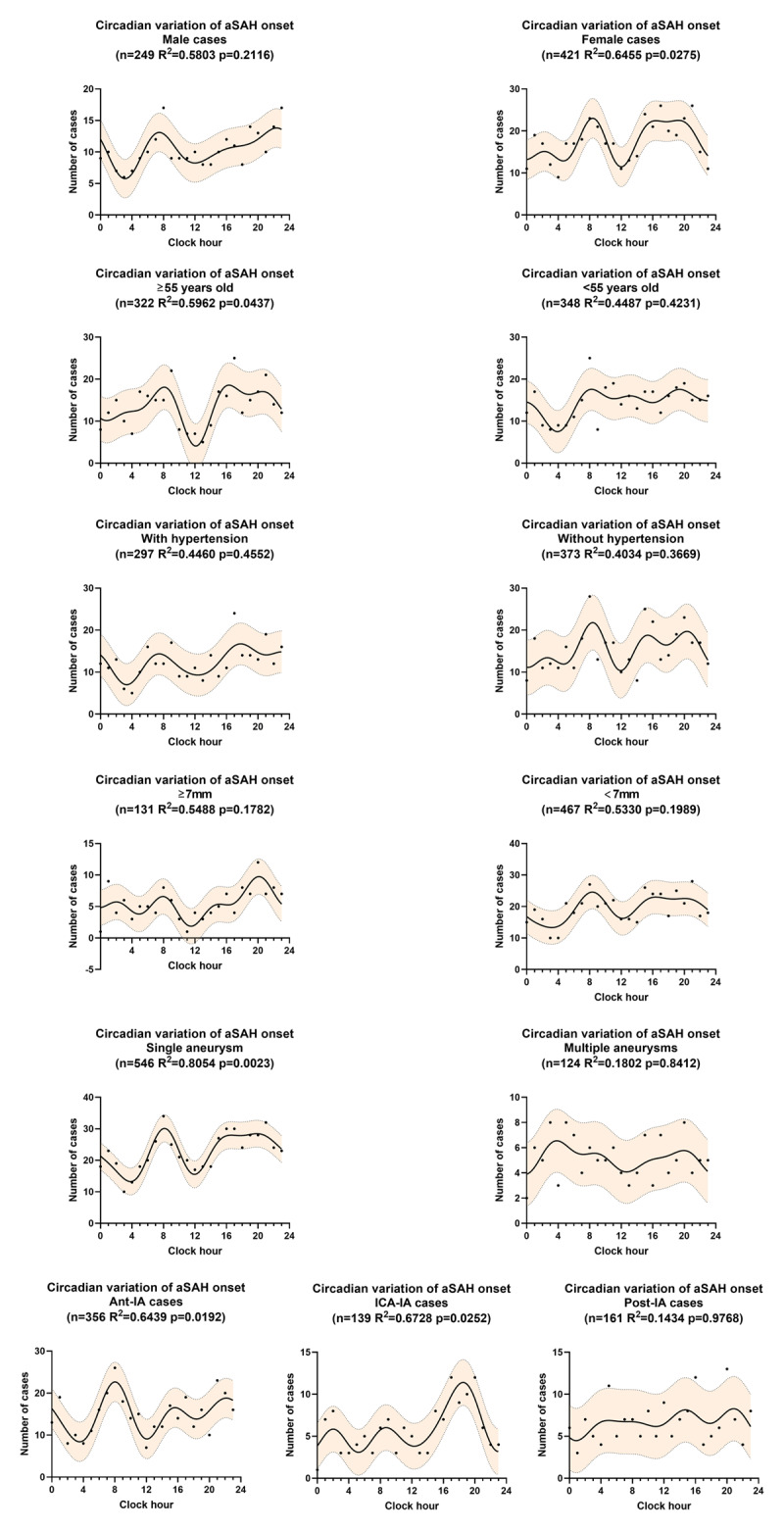
Subgroup analyses for circadian variation of aSAH onset, fitted by a four-harmonic Fourier model with periods of 24, 12, 8, and 6 hours. The solid line is the calculated best-fitting curve. The filled area represents 95% CI. aSAH, aneurysmal subarachnoid hemorrhage. Ant-IA, aneurysm in anterior circulation arteries after bifurcation of the internal carotid artery; ICA-IA, aneurysm in internal carotid artery and branches except for Ant-IA; Post-IA, aneurysm in posterior circulation artery.

Similar patterns were detected in the female patients (p = 0.0275), older patients (p = 0.0437), patients with a single aneurysm (p = 0.0023), Ant-IA group (p = 0.0192) and ICA-IA group (p = 0.0252) (***Figure 5***). The remaining subsets showed no significant circadian patterns. Subgroup analyses also identified diverse diurnal distributions of aSAH onset. The secondary peak was observed in 22:00–23:00 in male patients while this peak was observed in 16:00–20:00 in female patients. Younger patients (<55 years) showed no obvious midday trough and a later nocturnal trough (around 4:00) compared with older patients (nocturnal trough in 0:00–1:00). Ant-IA and ICA-IA patients showed different peak periods within a day, the former peaked around 8:00 while the latter peaked around 19:00.

## 4. Discussion

Our study systematically analyzed the chronobiological patterns including seasonal, monthly, weekly, and circadian distributions of aSAHs. Our results indicated that the occurrence of aSAH in central China exhibits obvious circannual and circadian patterns.

The incidence of aSAH is higher in winter and January/December compared to that in summer and July. Previous clinical studies reported similar results [5,11,15,16,17]. This circannual pattern could be associated with ambient temperature. Low daily mean, maximum, and minimum temperatures were associated with an increased rate of aSAH [11]. Another research also reported that temperature decline from the previous day is a trigger for the occurrence of aSAH [18]. Low temperature could induce physiological changes, such as increasing in sympathetic activity, peripheral vasoconstriction, and blood pressure [19]. These physiological changes during winter could lead to a surge in shear stress exerted on the aneurysm wall, triggering the occurrence of aSAH. The low temperature may also alter human behavior to a risky pattern, such as smoking indoors, heavy alcohol intake, and strenuous exercise [16]. These changes in human behavior could help to resist cold, but they are risk factors for aneurysm rupture. Moreover, the incidence of respiratory tract infection is higher during winter. Respiratory tract infection is also associated with a higher risk of SAH [20,21], which is probably due to increased inflammatory response and more common coughing and nose blowing in these patients [22].

Several studies revealed that ischemic stroke occurred more frequently after weekends [23,24]. The results in our study and other investigations [5,8] showed that there was no obvious weekly variation for the occurrence of aSAH. The day of the week could not trigger the onset of aSAH. However, we demonstrated that the onset of aSAH exhibits a significant circadian pattern, with apparently two peaks and two troughs. Among the 670 patients whose detailed onset time of aSAH was recorded, an evening trough appeared at 3:00–4:00, followed by a narrow morning peak around 8:00, a midday trough at 12:00–13:00, and a wide late afternoon peak from 16:00 to 20:00 in the chronobiological pattern. This circadian pattern is similar to the diurnal variation of blood pressure (BP), which may account for the circadian pattern of aSAH. BP exhibits a circadian rhythm in humans, that is mainly controlled by the innate circadian rhythm of the sympathetic nervous system. BP dips at night during rest, undergoes a steep increase in the morning, and peaks typically in the late afternoon [25]. The variation of BP is an important factor in the continuous expansion of the aneurysm [26]. The rupture site of IA is also reported to have higher pressure and shear stress oscillation [27], which are both associated with the variation of BP. Meanwhile, the reactivity of BP to physical activity varies with time of day, with the highest reactivity in the morning and a secondary rise in the afternoon [28]. During these periods, BP easily increases sharply. Besides, there is diurnal variation in most cardiovascular-related variables, such as heart rate, circulating catecholamines, platelet aggregability, peripheral arterial resistance and vasoconstriction, which also exhibit a peak in the morning [29]. Therefore, the variation of BP and other related variables, could contribute to the hemodynamic change and shear stress increase, triggering the occurrence of aSAH in corresponding periods.

Subsequent subgroup analyses identified that patients with different locations of aneurysms presented different circannual and circadian patterns. These results have not been reported by others. The risk of aneurysm rupture varies with location [1], indicating that factors such as vascular structure, constriction, shear stress, as well as the histopathology of aneurysm could be different among parental arteries. These confounding factors may influence the chronobiological patterns of aSAH. However, these factors are not assessable in our study. This hypothesis needs further confirmation.

Our analyses also identified several subtle discrepancies in circadian analyses among other subpopulations. Firstly, Male patients showed no obvious late afternoon peak compared with female patients. The prevalence of smoking and alcohol addiction was higher in Chinese men than that in women [30,31]. These factors, as well as different working statuses and estrogen level [32] between men and women may partially disturb the distribution of aSAH onset. Secondly, younger patients (<55 years) showed no obvious midday trough and a later nocturnal trough (around 4:00) compared with older patients (nocturnal trough in 0:00–1:00). The difference in sleeping, waking and siesta habits between them may account for the discrepancy. Siesta is reported to be associated with a lower risk of aneurysm rupture [33]. Thirdly, patients with multiple aneurysms showed no significant circadian pattern. Although patients with multiple aneurysms have more susceptible factors related to aneurysm growth and rupture comparing with single aneurysm [34,35], the huge gap in sample size between these two groups makes this result doubtful. A verification of this result is still needed. Finally, there were no significant results in the analyses of hypertension statues and aneurysmal size, though the peaks and nadirs were still recognizable. The reduced sample size in subgroup analyses may limit the statistical power.

Several epidemiological studies in western countries have explored the circadian pattern of aSAH onset. Feigin et al. [5] performed a study including 783 patients with SAH (75% of patients had proven aneurysm rupture as the cause for SAH) from three population-based studies and reported a trough incidence of aSAH at night and a peak incidence in the morning between 6:00 to 12:00. Temes et al. [6] conducted a study among 202 patients with aSAH and used the cosinor nonlinear least-squares analysis. They reported a significant morning peak from 7:00 to 8:00. However, these studies showed no obvious afternoon peak and did not perform subgroup analyses. In this study, we identified a bimodal circadian pattern (morning peak and late afternoon peak) of aSAH onset among the Chinese population by using a nonlinear Fourier model and also performed detailed subgroup analyses to assess potential influencing factors. Some Asian studies reported similar bimodal pattern [3,10,36]. The difference in chronobiological patterns between Asians and Europeans may due to diverse social habits and dietary patterns in different cultures. Asians prefer to consume hard liquor and salty food at dinner, which could contribute to the elevation in BP and account for the second peak of aSAH onset [37].

Overall, this study thoroughly identified the particular chronobiological patterns of aSAH onset in central China. Investigating the chronobiological patterns of aSAH onset could contribute to the development of new preventive strategies for this devastating disease. The modification of human behavior at a specific period may reduce the risk of aSAH onset, such as avoid exposure to low ambient temperatures in the winter and reduce the intake of salt and alcohol at dinner. Moreover, reducing the sudden increase in BP may also have benefits. A recent study suggested that the intake of long-term antihypertensive drugs at bedtime could improve the morning BP surge and reduce cardio- and cerebrovascular disease risk [38,39]. Analyses in the subgroups further identified that patients with different ages, sexes, and aneurysmal locations could present different chronobiological patterns. These findings could provide insight into triggering factors of aSAH onset, especially how aneurysms of different locations were triggered.

Our study has several limitations. Firstly, this study used a single-center retrospective design and extracted data from electronic medical records. These data could include bias as part of these data were based on patient recalls. Secondly, our data was based on patients admitted to one hospital. Thus, data from patients who failed to reach hospitals were missing, which would inevitably affect the accuracy of our results. Thirdly, several risk factors such as smoking and family history were not analyzed in this study due to a lack of information.

## 5. Conclusion

In summary, our study revealed that the onset of aSAH in central China exhibits clear chronobiological patterns. Our detailed subgroup analyses further revealed that differences in age, sex, and aneurysmal location could lead to different chronobiological patterns. The mechanism of these chronobiological patterns remains unclear and complex. Our findings could contribute to the development of new preventive strategies of aSAH and provide novel perspectives for further studies to investigate relationships between physiological factors, environmental factors, behavioral factors, dietary habits, and the occurrence of aSAH.

## Additional Files

The additional files for this article can be found as follows:

10.5334/gh.1117.s1 Supplementary Figures. Figures 1 to 2.

10.5334/gh.1117.s2 Supplementary Tables. Tables 1 to 5.
