# Supplementary figures and images for: Chronobiological Patterns of Aneurysmal Subarachnoid Hemorrhage in Central China
Source: Glob Heart. 2022 Apr 28;17(1):29. doi: 10.5334/gh.1117 (PMC9053527; doi:10.5334/gh.1117)

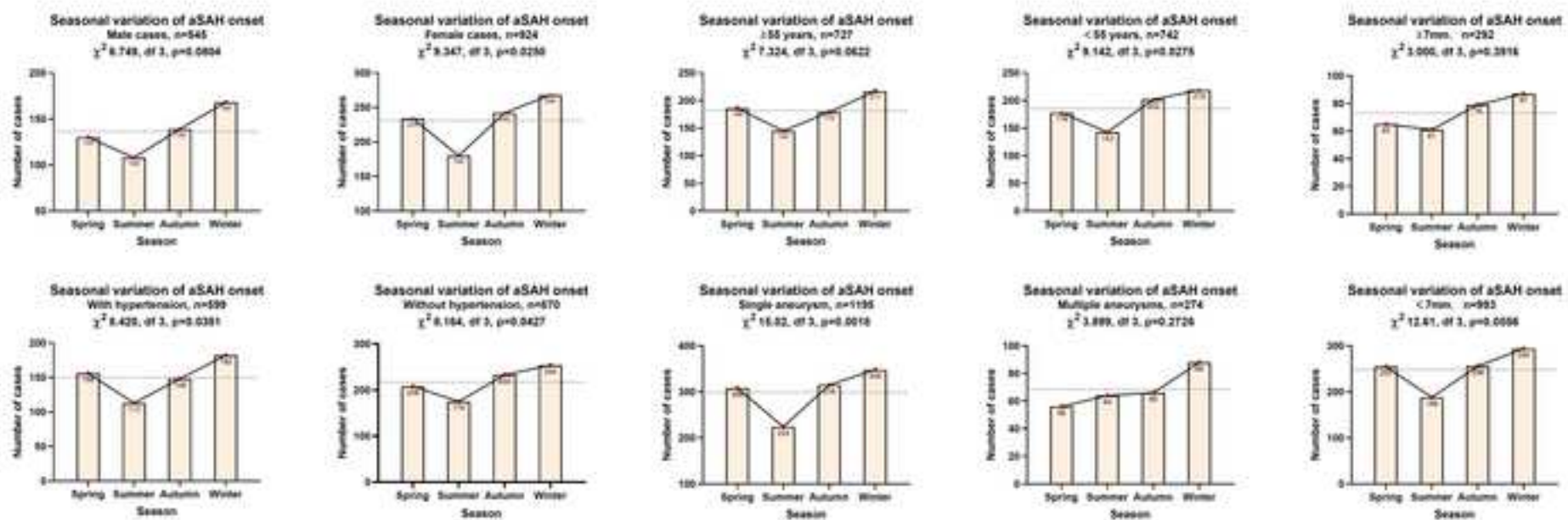

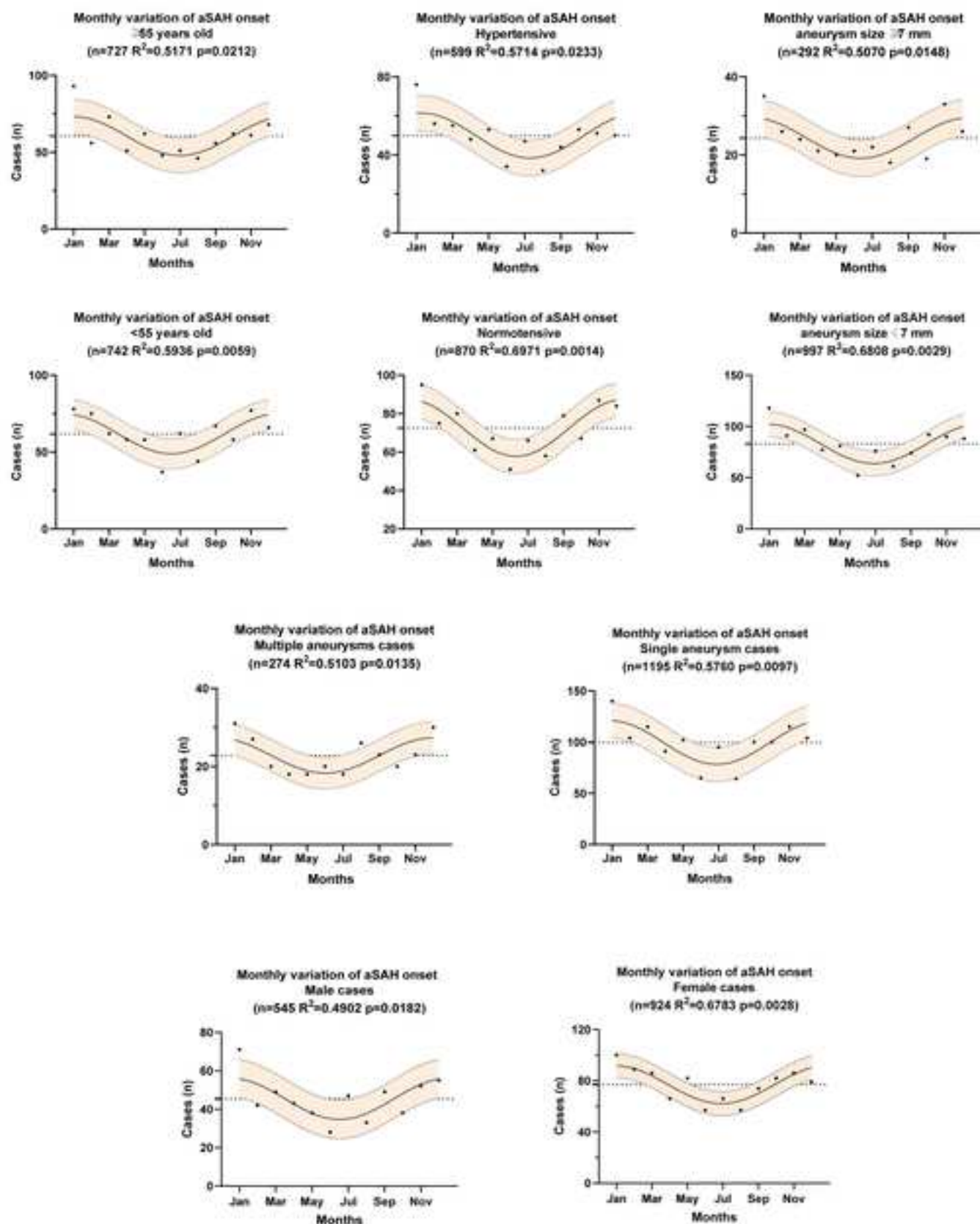

Supplement: Supplementary Figures. — Figures 1 to 2. [file gh-17-1-1117-s1.pdf]
