# Supplementary material for: Chronobiological Patterns of Aneurysmal Subarachnoid Hemorrhage in Central China
Source: Glob Heart. 2022 Apr 28;17(1):29. doi: 10.5334/gh.1117 (PMC9053527; doi:10.5334/gh.1117)
Supplement: Supplementary Tables. — Tables 1 to 5. [file gh-17-1-1117-s2.pdf]

**Supplementary Table 1 Characteristics of the study population for circannual and weekly variation analyses**

| Characteristics | All (n=1469) | Male (n=545) | Female (n=924) |
|---|---|---|---|
| Age (year), mean (SD) | 54.7±10.0 | 52.9±10.2 | 55.8±10.0 |
| Hypertension, n (%) | 599 (40.8) | 200 (33.3) | 399 (43.2) |
| Diabetes, n (%) | 53 (3.6) | 19 (3.5) | 34 (3.7) |
| Multiple aneurysms, n (%) | 274 (18.7) | 77 (14.1) | 197 (21.3) |
| Single aneurysm, n (%) | 1195 (81.3) | 468 (85.9) | 727 (78.7) |

**Supplementary Table 2 Summary characteristics of ruptured aneurysms in circannual and weekly variation analyses**

| Summary Statistic | All, n (%) | Male, n (%) | Female, n (%) |
|---|---|---|---|
| Size of ruptured aneurysm | | | |
| ≥ 7mm | 292 (22.7) | 108 (23.4) | 184 (22.2) |
| < 7mm | 997 (77.3) | 353 (76.6) | 644 (77.8) |
| Location of ruptured aneurysm | | | |
| Ant-IA | 733 (51.2) | 354 (66.5) | 379 (42.1) |
| ACA | 65 | 29 | 36 |
| AcoA | 413 | 210 | 203 |
| MCA | 255 | 115 | 140 |
| ICA-IA | 318 (22.2) | 68 (12.8) | 250 (27.7) |
| AChA | 14 | 6 | 8 |
| ICA | 292 | 59 | 233 |
| OA | 12 | 3 | 9 |
| Post-IA | 382 (26.6) | 110 (20.7) | 272 (30.2) |
| PCA | 16 | 5 | 11 |
| PcoA | 268 | 59 | 209 |
| AICA | 8 | 2 | 6 |
| PICA | 10 | 3 | 7 |
| VA/BA | 80 | 41 | 39 |

Note: n=number of cases; mm=millimeter; **Ant-IA=IA in anterior circulation arteries after bifurcation of the internal carotid artery**, ACA=anterior cerebral artery, ACoA=anterior

communicating artery, MCA=middle cerebral artery, **ICA-IA=IA in internal carotid artery and branches except for Ant-IA**, ICA=internal carotid artery, AChA=anterior choroidal artery, OA= ophthalmic artery, **Post-IA=IA in posterior circulation arteries**, PCoA=posterior communicating artery, PCA=posterior cerebral artery, AICA=anterior inferior cerebellar artery, PICA=posterior inferior cerebellar artery, VA=vertebral artery, BA=basilar artery.

**Supplementary Table 3 Characteristics of the study population for circadian variation analyses**

| Characteristics | All (n=670) | Male (n=249) | Female (n=421) |
|---|---|---|---|
| Age (year), mean (SD) | 54.4±10.2 | 52.7±9.988 | 55.4±9.976 |
| Hypertension, n (%) | 297 (44.3) | 102 (41.0) | 195 (46.3) |
| Diabetes, n (%) | 25 (3.7) | 10 (4.0) | 21 (5.0) |
| Multiple aneurysms, n (%) | 124 (18.5) | 36 (14.5) | 88 (20.9) |
| Single aneurysm, n (%) | 546 (81.5) | 213 (85.5) | 333 (79.1) |

**Supplementary Table 4 Summary characteristics of ruptured aneurysms in circadian variation analyses**

| Summary Statistic | All, n (%) | Male, n (%) | Female, n (%) |
|---|---|---|---|
| Size of ruptured aneurysm | | | |
| ≥ 7mm | 131 (21.9) | 46 (21.7) | 85 (22.0) |
| < 7mm | 467 (78.1) | 166 (78.3) | 301 (88.0) |
| Location of ruptured aneurysm | | | |
| Ant-IA | 356 (54.3) | 165 (67.6) | 191 (46.4) |
| ACA | 31 | 14 | 17 |
| AcoA | 192 | 96 | 96 |
| MCA | 133 | 55 | 78 |
| ICA-IA | 139 (21.2) | 29 (11.9) | 110 (26.7) |
| AChA | 5 | 3 | 2 |
| ICA | 128 | 25 | 103 |
| OA | 6 | 1 | 5 |
| Post-IA | 161 (24.5) | 50 (20.5) | 111 (26.9) |
| PCA | 7 | 3 | 8 |
| PcoA | 113 | 31 | 82 |
| AICA | 2 | 0 | 2 |
| PICA | 5 | 1 | 4 |
| VA/BA | 34 | 15 | 19 |

Note: n=number of cases; mm=millimeter; **Ant-IA=IA in anterior circulation arteries after bifurcation of the internal carotid artery**, ACA=anterior cerebral artery, ACoA=anterior communicating artery, MCA=middle cerebral artery, **ICA-IA=IA in internal carotid artery and**

**branches except for Ant-IA**, ICA=internal carotid artery, AChA=anterior choroidal artery, OA= ophthalmic artery, **Post-IA=IA in posterior circulation arteries**, PCoA=posterior communicating artery, PCA=posterior cerebral artery, AICA=anterior inferior cerebellar artery, PICA=posterior inferior cerebellar artery, VA=vertebral artery, BA=basilar artery.

**Supplementary Table 5 Weekly distribution of aSAH onset (subgroup analyses)**

| | Mon | Tue | Wed | Thu | Fri | Sat | Sun | p-value |
|---|---|---|---|---|---|---|---|---|
| ≥ 55 years | | | | | | | | |
| O | 114 | 98 | 99 | 108 | 101 | 98 | 109 | |
| E | 103.9 | 103.9 | 103.9 | 103.9 | 103.9 | 103.9 | 103.9 | |
| O/E | 1.10 | 0.94 | 0.95 | 1.04 | 0.97 | 0.94 | 1.05 | 0.9781 |
| < 55 years | | | | | | | | |
| O | 100 | 117 | 103 | 98 | 104 | 123 | 97 | |
| E | 106 | 106 | 106 | 106 | 106 | 106 | 106 | |
| O/E | 0.94 | 1.10 | 0.97 | 0.92 | 0.98 | 1.16 | 0.92 | 0.8390 |
| Hypertension | | | | | | | | |
| O | 81 | 79 | 86 | 77 | 84 | 93 | 99 | |
| E | 85.6 | 85.6 | 85.6 | 85.6 | 85.6 | 85.6 | 85.6 | |
| O/E | 0.95 | 0.92 | 1.00 | 0.90 | 0.98 | 1.09 | 1.16 | 0.9057 |
| Non-hypertension | | | | | | | | |
| O | 133 | 136 | 116 | 129 | 121 | 128 | 107 | |
| E | 124.3 | 124.3 | 124.3 | 124.3 | 124.3 | 124.3 | 124.3 | |
| O/E | 1.07 | 1.09 | 0.93 | 1.04 | 0.97 | 1.03 | 0.86 | 0.8590 |
| Single aneurysm | | | | | | | | |
| O | 177 | 180 | 168 | 166 | 167 | 175 | 162 | |
| E | 170.7 | 170.7 | 170.7 | 170.7 | 170.7 | 170.7 | 170.7 | |
| O/E | 1.04 | 1.05 | 0.98 | 0.97 | 0.98 | 1.02 | 0.95 | 0.9929 |
| Multiple aneurysms | | | | | | | | |
| O | 37 | 35 | 34 | 40 | 38 | 46 | 44 | |
| E | 39.1 | 39.1 | 39.1 | 39.1 | 39.1 | 39.1 | 39.1 | |
| O/E | 0.95 | 0.90 | 0.87 | 1.02 | 0.97 | 1.18 | 1.13 | 0.9586 |
| ≥ 7mm | | | | | | | | |
| O | 37 | 45 | 43 | 39 | 39 | 44 | 45 | |
| E | 41.7 | 41.7 | 41.7 | 41.7 | 41.7 | 41.7 | 41.7 | |
| O/E | 0.89 | 1.08 | 1.03 | 0.94 | 0.94 | 1.06 | 1.08 | 0.9922 |
| < 7mm | | | | | | | | |
| O | 152 | 135 | 138 | 142 | 145 | 149 | 136 | |
| E | 142.4 | 142.4 | 142.4 | 142.4 | 142.4 | 142.4 | 142.4 | |
| O/E | 1.07 | 0.95 | 0.97 | 1.00 | 1.02 | 1.05 | 0.96 | 0.9892 |
| Ant-IA | | | | | | | | |
| O | 101 | 107 | 111 | 104 | 107 | 108 | 95 | |
| E | 104.7 | 104.7 | 104.7 | 104.7 | 104.7 | 104.7 | 104.7 | |
| O/E | 0.96 | 1.02 | 1.06 | 0.99 | 1.02 | 1.03 | 0.91 | 0.9913 |
| ICA-IA | | | | | | | | |
| O | 47 | 40 | 36 | 48 | 48 | 52 | 47 | |

| | | | | | | | | |
|---|---|---|---|---|---|---|---|---|
| E | 45.4 | 45.4 | 45.4 | 45.4 | 45.4 | 45.4 | 45.4 | |
| O/E | 1.04 | 0.88 | 0.79 | 1.06 | 1.06 | 1.15 | 1.04 | 0.9136 |
| Post-IA | | | | | | | | |
| O | 61 | 62 | 53 | 49 | 44 | 51 | 58 | |
| E | 54 | 54 | 54 | 54 | 54 | 54 | 54 | |
| O/E | 1.13 | 1.15 | 0.98 | 0.91 | 0.81 | 0.94 | 1.07 | 0.8708 |

Note: O=observed frequency; E=Expected frequency; O/E=the ratio between observed frequency and expected frequency; p-value was calculated by Chi-square goodness-of-fit test.
